# Achievement of over 1.4 V photovoltage in a dye-sensitized solar cell by the application of a silyl-anchor coumarin dye

**DOI:** 10.1038/srep35888

**Published:** 2016-10-20

**Authors:** Kenji Kakiage, Hiroyuki Osada, Yohei Aoyama, Toru Yano, Keiji Oya, Shinji Iwamoto, Jun-ichi Fujisawa, Minoru Hanaya

**Affiliations:** 1 Environmental & Energy Materials Laboratory, ADEKA CORPORATION, 7-2-35 Higashiogu, Arakawa, Tokyo 116-8554, Japan; 2 Division of Molecular Science, Graduate School of Science and Technology, Gunma University, 1-5-1 Tenjin-cho, Kiryu, Gunma 376-8515, Japan

## Abstract

A dye-sensitized solar cell (DSSC) fabricated by using a novel silyl-anchor coumarin dye with alkyl-chain substitutes, a Br_3_^−^/Br^−^ redox electrolyte solution containing water, and a Mg^2+^-doped anatase-TiO_2_ electrode with twofold surface modification by MgO and Al_2_O_3_ exhibited an open-circuit photovoltage over 1.4 V, demonstrating the possibility of DSSCs as practical photovoltaic devices.

Dye-sensitized solar cells (DSSCs, Figs S1 and S2) have been investigated actively as practical photovoltaic cells of the next generation, because of their ease of fabrication, shorter energy and CO_2_ payback times, possibly flexible and colorful characteristics, and fine photovoltaic properties especially in low-light intensities and under scattered lights such as indoor conditions^1^,^2^,^3^. The light-to-electric energy conversion efficiency of DSSCs has been improved continuously by the development of the constituents of the cell^1^,^2^,^3^,^4^,^5^,^6^,^7^,^8^, and so far the efficiency has reached higher than 14%^9^. When considering the practical application of the DSSCs, the photovoltage is also an important photovoltaic property and the improvement of the photovoltage would extend the applicability of DSSCs^9^,^10^,^11^,^12^,^13^,^14^. The expectable photovoltage (*V*_exp_) in the DSSC depends on the energy gap between the quasi-Fermi level (approximately the energy level of the conduction-band edge, *E*_C.B._) of the metal-oxide electrode and the redox potential of the redox mediator in the electrolyte (Fig. S2), and therefore typical DSSCs made of the anatase-TiO_2_ electrode and the I_3_^−^/I^−^ redox mediator exhibit photovoltage lower than 0.9 V^1^,^2^,^3^,^4^.

Recently, we succeeded in obtaining a high open-circuit photovoltage (*V*_oc_) of 1.21 V in the DSSC by fabricating the cell with using a Mg^2+^-doped anatase-TiO_2_ (Mg-doped TiO_2_) electrode with negatively shifted *E*_C.B._ than the anatase-TiO_2_ electrode, a coumarin dye of **SFD-5** (Fig. 1a) possessing an alkoxysilyl group as an anchor moiety for a chemisorption to the TiO_2_ electrode, and an electrolyte with the Br_3_^−^/Br^−^ redox mediator which has more positive redox potential than the ordinary I_3_^−^/I^−^ redox mediator and Co^3+^/Co^2+^ complexes (Fig. S3)^13^. However, the expectable photovoltage of the cell was estimated to be ~1.5 V and there is still room for improvement, which will allow the usage of DSSCs as an alternative to the conventional dry cells and as a charging device for the rechargeable nickel-metal hydride batteries. An introduction of alkyl-chain substituents near to the silyl-anchor moiety in the coumarin dye is expected to improve the photovoltage by preventing the back electron transfer from the electrode to the redox electrolyte^15^,^16^,^17^,^18^. The development of surface modifications of the TiO_2_ electrode using wide bandgap metal oxides or insulators would also bring higher photovoltage by obstructing the back electron transfer^19^,^20^. In addition, the *E*_C.B._ of the Mg-doped TiO_2_ can be raised more by increasing the amount of Mg doped in TiO_2_ from the Mg/Ti atomic ratio of 0.10 to higher^21^. Thus, we newly designed and synthesized an alkoxysilyl coumarin dye of **ADEKA-3** (Fig. 1b), and succeeded in obtaining the photovoltage higher than 1.4 V by preparing Mg-doped TiO_2_ with larger Mg composition, applying twofold metal-oxide surface modification to the Mg-doped TiO_2_ electrodes by MgO and Al_2_O_3_, and adding water to the electrolyte solution of Br_3_^−^/Br^−^ redox mediator with using the advantage of the durability of the alkoxysilyl-dye adsorbed electrodes to water.

## Results and Discussion

In **ADEKA-3**, the introduction of alkyl-chain substituents was performed by linking hexyl-thiophene rings to the coumarin moiety. A methyl group was also added to the coumarin moiety to prevent the co-planar arrangement of the coumarin moiety and the thiophene ring, which will produce a heightening of the HOMO level of the dye through the extension of the coumarin π system to the thiophene ring. The alkoxysilyl coumarin dye of **ADEKA-3** exhibited similar UV-visible absorption spectra to **SFD-5** in solutions, and a major absorption band of **ADEKA-3** solution assignable to the π-π^*^ transition was observed in visible region between 350 and 500 nm. The maximum molar absorption coefficient (ε_max_) at λ_max_ was evaluated to be 48,700 at 415 nm (Fig. S4). The energy levels of HOMO and LUMO were estimated to be 1.18 V and −1.12 V *vs.* NHE, respectively, for **ADEKA-3** (Table S1). The HOMO level is more positive than the redox potential of ~0.9 V *vs.* NHE of the Br_3_^−^/Br^−^ redox^10^,^13^,^22^, thus providing thermodynamic driving force for the dye regeneration reaction by the electron transfer from the Br_3_^−^/Br^−^ redox mediator to the oxidized dye formed through the light-excited-electron injection to the TiO_2_ electrode. The reliability of the relative positions of HOMO and LUMO levels were supported by the molecular orbital calculations for **SFD-5** and **ADEKA-3** (Figs S7 and S8).

**Figure 2:**
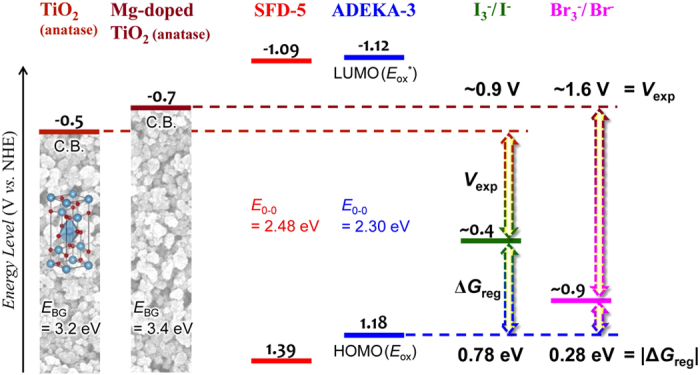
Energy levels. Schematic energy diagram of the DSSC composed of the TiO_2_ and Mg-doped TiO_2_ (Mg/Ti = 0.20, atomic ratio) electrodes, the silyl-anchor coumarin dyes of **SFD-5** and **ADEKA-3**, and the redox electrolytes of I_3_^−^/I^−^ and Br_3_^−^/Br^−^ mediators.

The Mg-doped TiO_2_ crystalline nanoparticles with an increased Mg/Ti atomic ratio to 0.20 were synthesized by the solvothermal method^13^,^21^. As the reference to the Mg-doped TiO_2_, anatase-TiO_2_ nanoparticles without Mg-doping (undoped-TiO_2_) were also synthesized by the same method. The single phase of anatase structure was confirmed for the Mg-doped TiO_2_ crystalline nanoparticles by X-ray diffraction (XRD) experiments, and the particle size was estimated to be ~25 nm by using the Scherrer equation (Figs S9 and S10). The band gap of the Mg-doped TiO_2_ was evaluated to be 3.4 eV by a Tauc plot of the diffuse reflectance spectrum (Figs S11 and S12), which is 0.2 eV larger than that of the undoped-TiO_2_ consistently with the negative shift of the *E*_C.B._ by the Mg-doping^21^,^23^. Energy levels of the Mg-doped TiO_2_, **ADEKA-3**, and the Br_3_^−^/Br^−^ redox mediator are drawn schematically in Fig. 2, which shows the suitability of **ADEKA-3** as the sensitizing dye in the cell system with the Mg-doped TiO_2_ and the Br_3_^−^/Br^−^ redox mediator.

The results of *J*-*V* measurements performed in this work are listed in Table 1 and shown in Fig. S13. The measurements were performed under AM-1.5G one sun illumination (100 mW cm^−2^). To check the performance of **ADEKA-3** as a photosensitizer, *J*-*V* measurements were carried out for the cells sensitized by **SFD-5** and **ADEKA-3** as Entry 1 and 2, respectively, at 25 °C with using the TiO_2_ electrode without Mg-doping and a Br_3_^−^/Br^−^ redox electrolyte solution (Electrolyte A: See Methods for the compositions of electrolytes used in this work.). The cell sensitized by **ADEKA-3** exhibited 0.1 V higher *V*_oc_ and smaller short-circuit photocurrent density (*J*_sc_) than the cell sensitized by **SFD-5**, and light-to-electric energy conversion efficiencies (*η*) of these cells were almost the same. Since the dark current was smaller in the **ADEKA-3**-sensitized cell than the **SFD-5**-sensitized cell (Fig. S14), the increase of *V*_oc_ in the **ADEKA-3**-sensitized cell is considered to be brought by the hexyl-chain substituents introduced in **ADEKA-3**, which are working as the suppressor for preventing the back electron transfer from the TiO_2_ electrode to the Br_3_^−^/Br^−^ redox electrolyte by covering the naked surface of the TiO_2_ electrode between the adsorbed dye molecules^15^,^16^,^17^,^18^. In **ADEKA-3**, the HOMO level is higher in energy than that of **SFD-5** by 0.21 eV, and thus the energy gap between the HOMO level and the redox potential of the Br_3_^−^/Br^−^ redox mediator is smaller than that for **SFD-5** (Fig. 2 and Table S1). The incident monochromatic photon-to-current conversion efficiencies (*IPCE*) were observed to tend to be lower in the **ADEKA-3**-sensitized cell than the **SFD-5**-sensitized cell (Fig. S15), and thus the smallness of the energy gap is considered as the reason for the lower *J*_sc_ value in the **ADEKA-3**-sensitized cell, which produced the delay of the dye regeneration reaction proceeding through the electron transfer from the redox mediator in the electrolyte solution to the dye in the oxidized state^10^,^22^.

Since **ADEKA-3** was ascertained as an effective dye for producing high photovoltage, the Mg-doped TiO_2_ electrode was applied to the cell sensitized by the dye as Entry 3. The cell exhibited the *V*_oc_ of 1.23 V, which is about 20% higher than that of the cell using the TiO_2_ electrode without Mg-doping. The increase of the *V*_oc_ is considered to be due to the higher *E*_C.B._ of the Mg-doped TiO_2_ than that of the TiO_2_ without Mg-doping. For a further increment of the photovoltage in the cell, we examined surface modifications of the Mg-doped TiO_2_ electrode by wide bandgap metal oxides of MgO and by Al_2_O_3_ following the MgO modification (MgO + Al_2_O_3_) as Entries 4 and 5, respectively. The surface modification by MgO was confirmed to be effective also in the present cell system in improving the photovoltage, and the improvement is understood as the result of the negative shift of the *E*_C.B._ of the Mg-doped TiO_2_ by the MgO modification (Fig. S16)^20^. More efficient improvement was observed by the twofold surface modification with MgO and Al_2_O_3_. The Al_2_O_3_ modification was confirmed not to affect the *E*_C.B._ of the MgO-modified Mg-doped TiO_2_ by the UV-visible spectra (Fig. S16), and the modification is thought to form a blocking layer on the surface of the Mg-doped TiO_2_ electrode suppressing the back electron transfer from the electrode to the redox mediator in the electrolyte solution^19^,^20^.

**Figure 3:**
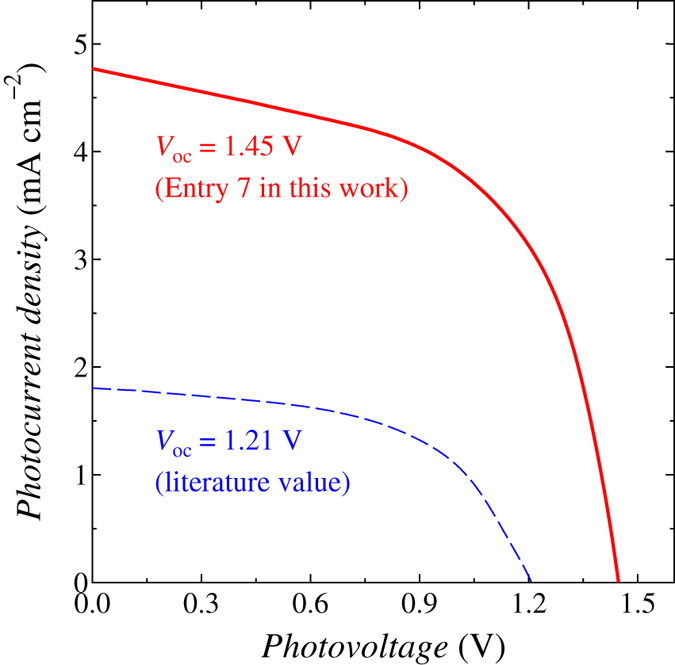
*J*-*V* characteristics. *J*-*V* properties of the **ADEKA-3**-photosensitized cell with the highest *V*_oc_ of 1.45 V (Entry 7 in Table 1) (solid line) and the reported **SFD-5**-photosensitized cell with the *V*_oc_ of 1.21 V (dashed line)^13^ under the simulated one sun irradiation (AM-1.5G, 100 mW cm^−2^) at 25 °C.

In DSSCs, photovoltage is known to be increased by the addition of compounds having coordination ability to the surface of TiO_2_ electrodes, such as 4-*tert*-butylpyridine (TBP), to electrolyte solutions which shift the *E*_C.B._ negatively through the coordination. We examined the addition of 4-methylpyridine (MP) and 4-trimethylsilylpyridine (TMSP) to the Br_3_^−^/Br^−^ redox electrolyte solution^24^, and prepared Electrolyte B with an experimentally optimized composition for high photovoltage. By using the electrolyte solution as Entry 6, the *V*_oc_ was increased slightly and reached to 1.39 V. As an additive to the electrolyte solution for the improvement of the photovoltage, water is expected to be effective because of its high coordination ability owing to lone pairs on the oxygen atom and small molecular size^25^,^26^,^27^. However, the addition of water to electrolyte solutions causes the elimination of sensitizing dyes from the TiO_2_ electrodes generally in the case of conventional carboxy-anchor dyes, and the application of electrolyte solutions containing water has been rather limited^28^. On the other hand, alkoxysilyl dyes chemisorb the TiO_2_ electrodes by forming Si-O-Ti bonds through the condensation reaction between the alkoxysilyl groups and the hydroxy groups on the TiO_2_ surface, and the dye adsorbed electrodes have quite high durability to solvents, e.g. nitrile, water, and mixture of them^7^,^29^,^30^,^31^. Thus, we attempted to use a Br_3_^−^/Br^−^ redox electrolyte solution containing water with the concentration of 0.10 M (Electrolyte C) as Entry 7. By the addition of water to the electrolyte, the *V*_oc_ was improved actually to 1.45 V. The addition of water also brought about a decrement of the photocurrent to the cell, but the *η* was still to be ~4% (Tables 1 and S2, and Figs 3 and S13). And further, the *V*_oc_ reached 1.50 V by lowering the cell temperature to 5 °C (Entry 8) as the result of a possible rise of the *E*_C.B._ and a deceleration of the back electron transfer reaction^32^. To the best of our knowledge, the observed *V*_oc_ of 1.45 V at an ordinary temperature is the highest ever reported for DSSCs with a single-cell structure (Table S3)^7^,^8^,^9^,^10^,^11^,^13^,^14^,^17^,^21^,^33^,^34^.

## Conclusions

We succeeded in producing the photovoltage over 1.4 V with a reasonably high conversion efficiency close to 4% in the DSSC by using the alkoxysilyl-anchor coumarin dye of **ADEKA-3**, the Mg-doped TiO_2_ electrode with the twofold surface modification by MgO and Al_2_O_3_, and the Br_3_^−^/Br^−^ redox electrolyte solution containing water. The observed *V*_oc_ is higher than those of other types of solar cells (Table S4), and is comparable to that of a conventional dry cell^5^. The achievement of such a high photovoltage, which is owing to the surpassing property of a silyl-anchor dye as a sensitizing dye for DSSCs, demonstrates the possibility of DSSCs as practical photovoltaic devices.

## Methods

### Device fabrication

Preparation procedures of the dye (**SFD-5** or **ADEKA-3**)-adsorbed TiO_2_ electrodes used in the cells were described in Supplementary Information. When applying the MgO surface modification to the Mg-doped TiO_2_ electrodes, the electrodes before the dye adsorption were immersed into a 50 mM 2-propanol solution of Mg(OC_2_H_5_)_2_ at 25 °C for 1 h, rinsed in ethanol, and then calcined in air at 490 °C for 30 min^20^. In the twofold surface modification with MgO and Al_2_O_3_, the MgO-modified Mg-doped TiO_2_ electrodes were immersed into a 30 mM 2-propanol solution of Al[OCH(CH_3_)_2_]_3_ at 25 °C for 45 min, rinsed in ethanol, and then calcined in air at 490 °C for 1 h^20^. We used electrochemical cells of an open sandwich type through this work for photovoltaic measurements. A Pt-treated FTO-coated glass plate which was prepared by the rf magnetron sputtering of Pt and the reported H_2_PtCl_6_ treatment^35^ was employed as the counter electrode. Three Br_3_^−^/Br^−^ redox electrolyte solutions were used as the electrolytes: Electrolyte A) 0.03 M Br_2_ + 0.65 M 1-*n*-butyl-3-methylimidazolium bromide (BMImBr) + 0.20 M tetra-*n*-pentylammonium bromide (TPABr) + 0.07 M 4-*tert*-butylpyridine (TBP) + 0.07 M guanidinium thiocyanate (GuSCN) in acetonitrile (AN)/valeronitrile (VN)/ethylene carbonate (EC)/tetrahydrofuran (THF) (4:3:2:1 in volume)^13^, Electrolyte B) 0.03 M Br_2_ + 0.65 M BMImBr + 0.20 M TPABr + 0.05 M TBP + 0.01 M 4-methylpyridine (MP) + 0.02 M 4-trimethylsilylpyridine (TMSP) + 0.07 M GuSCN in AN/VN/EC/THF (4:3:2:1 in volume), and Electrolyte C) 0.03 M Br_2_ + 0.65 M BMImBr + 0.20 M TPABr + 0.05 M TBP + 0.01 M MP + 0.02 M TMSP + 0.07 M GuSCN + 0.10 M H_2_O in AN/VN/EC/THF (4:3:2:1 in volume) (Fig. S17). The Mg-doped TiO_2_ or TiO_2_ electrode sensitized by **SFD-5** or **ADEKA-3**, the counter electrode, and a polyethylene film spacer of 30 μm thick were assembled, and one of the Br_3_^−^/Br^−^ redox electrolyte solutions was injected into the space between the electrodes (Fig. S18).

### Photovoltaic measurements

The photovoltaic performances of the fabricated DSSCs were assessed from the *IPCE* spectra and the *J*-*V* properties of the cells with maintaining the aperture area of the cells to be 1.00 × 1.00 cm^2^ by the use of a square black shade mask. The *IPCE* spectra were obtained by using a monochromatic light source of SM-25 (Bunkoukeiki) and an electrometer of R8240 (Advantest) at 25 °C. The *J*-*V* properties were measured by using a solar simulator with Class AAA of OTENTO-SUN III (Bunkoukeiki) and a source meter of R6240A (Advantest) under the simulated sunlight irradiation of AM-1.5G one sun condition (100 mW cm^−2^) at 25 or 5 °C. The details were described in Supplementary Information.

### Molecular orbital calculation

We optimized the molecular structures and calculated the energy levels of frontier orbitals and others for the alkoxysilyl-anchor coumarin dyes on the Gaussian 09 program package by using a density functional theory (DFT)^36^. The details were described in Supplementary Information.

## Additional Information

**How to cite this article**: Kakiage, K. *et al*. Achievement of over 1.4 V photovoltage in a dye-sensitized solar cell by the application of a silyl-anchor coumarin dye. *Sci. Rep.* **6**, 35888; doi: 10.1038/srep35888 (2016).

## Supplementary Material

Supplementary Information

## Figures and Tables

**Figure 1:**
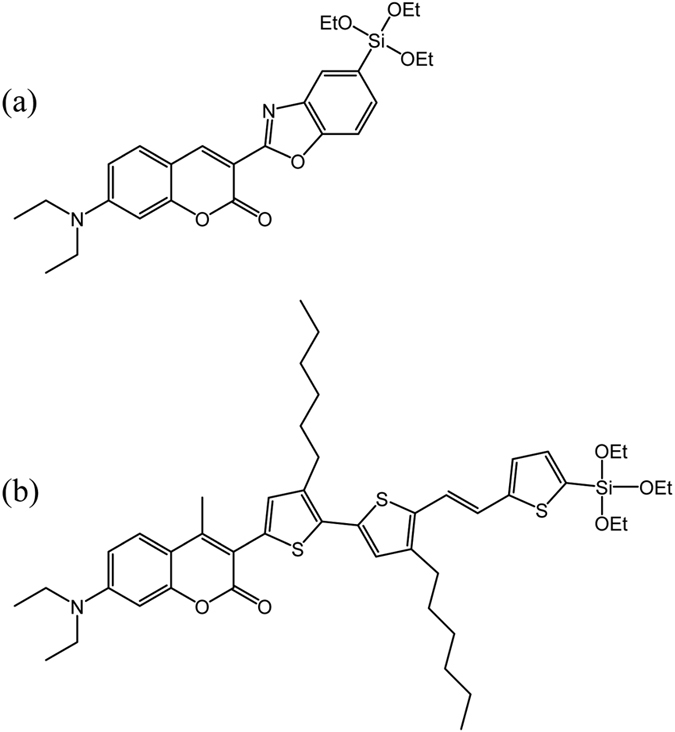
Sensitizing dyes. Molecular structures of silyl-anchor coumarin dyes: (**a**) **SFD-5** and (**b**) **ADEKA-3**.

**Table 1:** Photovoltaic data.

Entry	Dye	Electrode	Surface Modification	Electrolyte	Temp.	*J*_sc_ (mA cm^−2^)	*V*_oc_ (V)	*FF*	*η* (%)
1	**SFD-5**	TiO_2_	none	A	25 °C	6.16	0.96	0.53	3.1
2	**ADEKA-3**	TiO_2_	none	A	25 °C	5.21	1.05	0.54	3.0
3	**ADEKA-3**	Mg-doped TiO_2_	none	A	25 °C	5.11	1.23	0.56	3.5
4	**ADEKA-3**	Mg-doped TiO_2_	MgO	A	25 °C	5.02	1.31	0.56	3.7
5	**ADEKA-3**	Mg-doped TiO_2_	MgO + Al_2_O_3_	A	25 °C	4.90	1.37	0.56	3.8
6	**ADEKA-3**	Mg-doped TiO_2_	MgO + Al_2_O_3_	B	25 °C	5.10	1.39	0.55	3.9
7	**ADEKA-3**	Mg-doped TiO_2_	MgO + Al_2_O_3_	C	25 °C	4.77	1.45	0.56	3.9
8	**ADEKA-3**	Mg-doped TiO_2_	MgO + Al_2_O_3_	C	5 °C	4.41	1.50	0.54	3.7

Photovoltaic parameters of the cells with the alkoxysilyl-anchor coumarin dye of **SFD-5** or **ADEKA-3**, with the TiO_2_ or Mg-doped TiO_2_ (Mg/Ti = 0.20, atomic ratio) electrode, without or with the surface modification of the Mg-doped TiO_2_ electrode, and with the Br_3_^−^/Br^−^ redox electrolyte solution of Electrolyte A, B, or C under the illumination of the simulated sunlight (AM-1.5G, 100 mW cm^−2^) at 25 or 5 °C: short-circuit photocurrent density (*J*_sc_), open-circuit photovoltage (*V*_oc_), fill factor (*FF*), and light-to-electric energy conversion efficiency (*η*).
