# Supplementary material for: Achievement of over 1.4 V photovoltage in a dye-sensitized solar cell by the application of a silyl-anchor coumarin dye
Source: Sci Rep. 2016 Oct 20;6:35888. doi: 10.1038/srep35888 (PMC5071898; doi:10.1038/srep35888)
Supplement: Supplementary Information [file srep35888-s1.pdf]

Supplementary Information

Achievement of over 1.4 V photovoltage in a dye-sensitized solar cell by the application of a silyl-anchor coumarin dye

Kenji Kakiage[1], Hiroyuki Osada[1], Yohei Aoyama[1], Toru Yano[1], Keiji Oya[1], Shinji Iwamoto[2], Jun-ichi Fujisawa[2], Minoru Hanaya[2]

[1]Environmental & Energy Materials Laboratory, ADEKA CORPORATION, 7-2-35 Higashiogu, Arakawa, Tokyo 116-8554, Japan.

[2]Division of Molecular Science, Graduate School of Science and Technology, Gunma University, 1-5-1 Tenjin-cho, Kiryu, Gunma 376-8515, Japan.

Corresponding authors

Minoru Hanaya[2] (mhanaya@gunma-u.ac.jp)

Toru Yano[1] (yanotoru@adeka.co.jp)

Supplementary Information

a) *Synthesis of ADEKA-3*:

All reagents and solvents were obtained from commercial sources and used without further purification unless otherwise noted.

A mixture of **1** (1.000 g, 0.00156 mol), **2** (0.850 g, 0.00311 mol), **3** (0.042 g, 0.000046 mol), **4** (0.056 g, 0.00019 mol), **5** (0.600 g, 0.00467 mol), and **6** (15.0 mL) was stirred at 120 °C under argon for 3.5 h. Chloroform and water were added in the reaction. Filtration with celite, oil/water separation, column chromatography (ODS, MeOH : water = 9 : 1), and drying under vacuum at 40 °C for 5 h were performed to give **ADEKA-3** (0.181 g, yield = 14%) as an orange oil. ^{1}H NMR ($CDCl_3$, 400 MHz) δ_H 7.45 (d, 1H), 7.33 (d, 1H), 7.13 (d, 1H), 7.11 (s, 1H), 6.99 (d, 1H), 6.92 (s, 1H), 6.88 (s, 1H), 6.60 (dd, 1H), 6.50 (d, 1H), 3.91 (q, 6H), 3.42 (q, 4H), 2.81 (m, 2H), 2.65 (m, 2H), 2.44 (s, 3H), 1.67 (m, 4H), 1.35 (m, 21H), 1.20 (m, 6H), 0.90 (t, 6H).

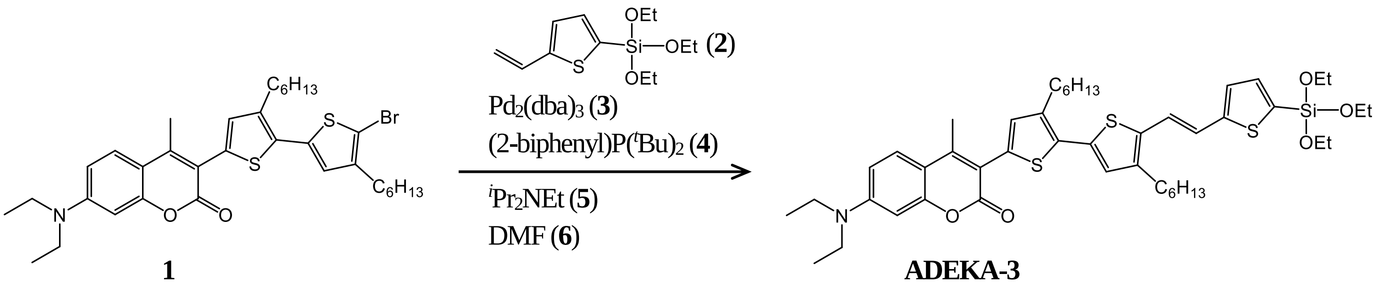

1 **ADEKA-3**

b) *Spectral and Electrochemical Measurements*:

The NMR spectra were obtained by using a JEOL JNM-ECA 400 Spectrometer. Chemical shifts were reported as δ units (ppm) relative to the standard material ($SiMe_4$). The UV-visible absorption spectra of **SFD-5** in an acetonitrile solution and **ADEKA-3** in a toluene solution at 25 °C were recorded on a JASCO V-650 UV-Vis Spectrophotometer, and an integrating sphere was equipped to the spectrophotometer for the measurements of the two photosensitizing dyes adsorbed on the TiO_2 electrodes. Cyclic voltammetry measurements were carried out at 25 °C in a three-electrode electrochemical cell on an ALS/CH Instruments Electrochemical Analyzer Model 600C to determine the oxidation potentials of the two silyl-anchor coumarin

dyes. Pt, Pt, and Ag/Ag^+ were employed as working, counter, and reference electrodes, respectively. The supporting electrolyte was 0.10 M tetrabutylammonium perchlorate (TBAP) in CH_2Cl_2 and the scan rate was set to be 100 mV s^{-1}. The potential of the reference electrode is 0.43 V versus normal hydrogen electrode (NHE) and was calibrated with ferrocene.

c) *Syntheses and Measurements of Mg-Doped TiO_2 (Mg/Ti = 0.20, atomic ratio) and TiO_2 Powders*:

Nanoparticles of the Mg-doped TiO_2 with a Mg/Ti atomic ratio of 0.20 were synthesized by the solvothermal method[1,2]. Titanium tetraisopropoxide (24.2 g), magnesium acetate tetrahydrate (3.66 g), and 1,4-butanediol (100 mL) were added to an autoclave. The atmosphere in the autoclave was replaced with N_2 gas, and then the assembly was heated at 300 °C for 2 h. After cooling, the solid product dispersed in the organic solvent was collected by a centrifugation, then washed in acetone and air-dried. The obtained powder was annealed in air at 450 °C for 30 min. Nanoparticles of the TiO_2 without Mg-doping were also synthesized by the same method.

X-ray diffraction (XRD) experiments of the Mg-doped TiO_2 and TiO_2 powders were carried out at room temperature with a Rigaku RINT 2200 X-ray diffractometer by using CuKα radiation. The diffraction angle was corrected by using a crystalline Si powder as an external standard. The crystalline nanoparticle sizes of the Mg-doped TiO_2 and TiO_2 powders were estimated from the diffraction peak widths by using Scherrer equation[3-5],

$$D = (0.9\,\lambda) / (B \cos \theta_B)$$

where D is the particle size of the nanocrystallites, λ is the X-ray wavelength and θ_B is the Bragg angle. The line broadening, B, is measured from the extra peak width at half the peak height and is obtained from the Warren formula: $B^2 = B_M{}^2 - B_S{}^2$, where B_M is the measured peak full width in radians at half peak height and B_S is the corresponding width of a peak of a standard material (Si crystal). The band gaps of the Mg-doped TiO_2 and TiO_2 powders were evaluated by the tauc plots ($[h\nu F(R_\infty)]^{1/2}$-$h\nu$ curves) of the UV-visible diffuse reflectance spectra which were recorded on a JASCO V-650 UV-Vis Spectrophotometer at 25 °C[6-8].

d) *Preparation of Mg-Doped TiO_2 and TiO_2 Electrodes with Photosensitizing Dyes*:

To prepare the working electrodes with a thin layer of the Mg-doped TiO_2 for DSSCs, F-doped SnO_2 (FTO)-coated glass plates (25 × 50 mm^2, 9-11 Ω $sq.^{-1}$; Asahi Glass) were used as the current collector. As

pre-treatments, the FTO-coated glass plates were UV-O_3 treated for 30 min, immersed into a 80 mM $TiCl_4$ aqueous solution at 75 °C for 1 h, washed with water and ethanol, and calcined in air at 300 °C for 30 min, and then immersed into a 100 mM $Nb(OC_4H_9)_5$ ethanol solution at 25 °C for 1 h, washed with ethanol, and calcined in air at 450 °C for 30 min[9,10]. On the UV-O_3, $TiCl_4$, and $Nb(OC_4H_9)_5$-treated FTO-coated glass plates, the thin layer of the Mg-doped TiO_2 was coated by a screen-printing method using a paste prepared by mixing the synthesized Mg-doped TiO_2 powder, water, nitric acid, and polyethylene glycol with homogenizers. The Mg-doped TiO_2 electrodes were then sintered in air at 490 °C for 1 h. The FE-SEM observation using a JEOL JSM-6330F field emission-scanning electron microscope with the operating voltage of 15.0 kV was performed for the Mg-doped TiO_2 electrode (Fig. S19), the thickness of the semitransparent porous thin layer of the Mg-doped TiO_2 was estimated to be ~5 μm by the cross section observation of the electrodes. The anatase-TiO_2 powder without Mg-doping was used as a reference to the Mg-doped TiO_2, and the working electrode with the TiO_2 powder was prepared in the same way as the Mg-doped TiO_2 electrode. Adsorption of the photosensitizing dyes of **SFD-5** and **ADEKA-3** on the electrodes was carried out by immersing the electrodes in toluene solutions with 3.0×10^{-4} M photosensitizing dyes and 1.0×10^{-3} M coadsorbent of isooctyltriethoxysilane (Gelest, Inc.) at 25 °C for 20 h. Before the immersion in the dye solutions, the electrodes were treated with UV-O_3, heated in air at 100 °C for 5 h, and then cooled to 80 °C to eliminate excess adsorbed water on the Mg-doped TiO_2 and TiO_2 surfaces for the efficient dye adsorption.

e) *Photovoltaic Measurements*:

The photovoltaic performances of the fabricated DSSCs were assessed from the incident monochromatic photon-to-current conversion efficiency (*IPCE*) spectra and the photocurrent-voltage (*J-V*) properties of the cells with maintaining the aperture area of the cells to be 1.00×1.00 cm^2 by the use of a square black shade mask. The *IPCE* spectra were obtained by using a monochromatic light source of SM-25 (Bunkoukeiki) and an electrometer of R8240 (Advantest) at 25 °C. The *J-V* properties were measured by using a solar simulator with Class AAA of OTENTO-SUN III (Bunkoukeiki) and a source meter of R6240A (Advantest) under the simulated sunlight irradiation of AM-1.5G one sun condition (100 mW cm^{-2}) at 25 °C. A low temperature measurement at 5 °C was also carried out. The power of the simulated sunlight was calibrated by the use of a reference Si photodiode for DSSCs of BS-520 (Bunkoukeiki). The *J-V* properties were obtained by applying an external bias to the cells and measuring the generated photocurrent with the source meter. The voltage step and

delay time for the photocurrent measurements were set to be 5 mV and 80 ms, respectively.

$IPCE$ (%) = {(1240 [V nm] × J_{ph} [mA cm^{-2}]) / (λ [nm] × I_m [mW cm^{-2}])} × 100

J_{ph} is the short-circuit photocurrent density for the monochromatic light irradiation. λ and I_m are the wavelength and the intensity of the monochromatic light, respectively.

η (%) = {(J_{sc} [mA cm^{-2}] × V_{oc} [V] × FF) / I_s [mW cm^{-2}]} × 100

The overall light-to-electric energy conversion efficiency (η) of the DSSC is determined by the short-circuit photocurrent density (J_{sc}), the open-circuit photovoltage (V_{oc}), the fill factor (FF) [= P_{max} / (J_{sc} × V_{oc})] of the cell and the intensity of the incident simulated sunlight (I_s). P_{max} is the product of J_{max} and V_{max}, that is photocurrent density and photovoltage at the voltage where the power output of the cell is maximal.

f) *Molecular Orbital (MO) Calculation*:

We optimized the molecular structures and calculated the energy levels of frontier orbitals and others for the alkoxysilyl-anchor coumarin dyes (**SFD-5** and **ADEKA-3**) on the Gaussian 09 program package by using a density functional theory (DFT)[11]. A Becke's three-parameter hybrid functional with the LYP correlation functional (B3LYP) was employed together with 6-31+G(d,p) basis set[12,13]. Geometry optimizations and calculations of electronic properties of the dyes were performed without any symmetry constraint in the gas phase and by assuming the target molecules to be isolated. Calculated molecular orbitals were visualized by using the GaussView 5.

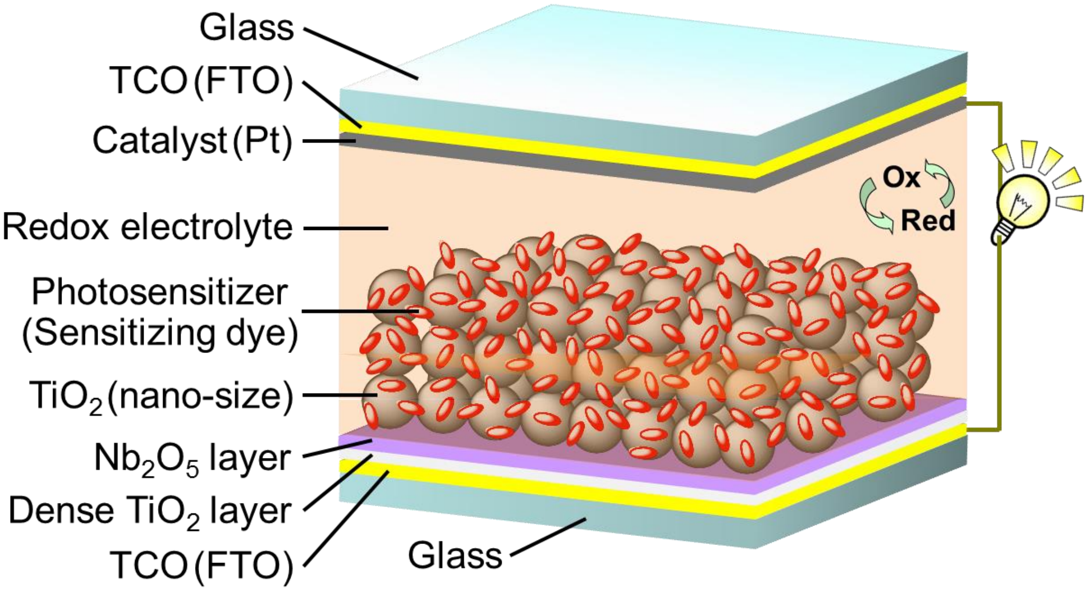

Fig. S1 Schematic illustration of the structure for a DSSC.

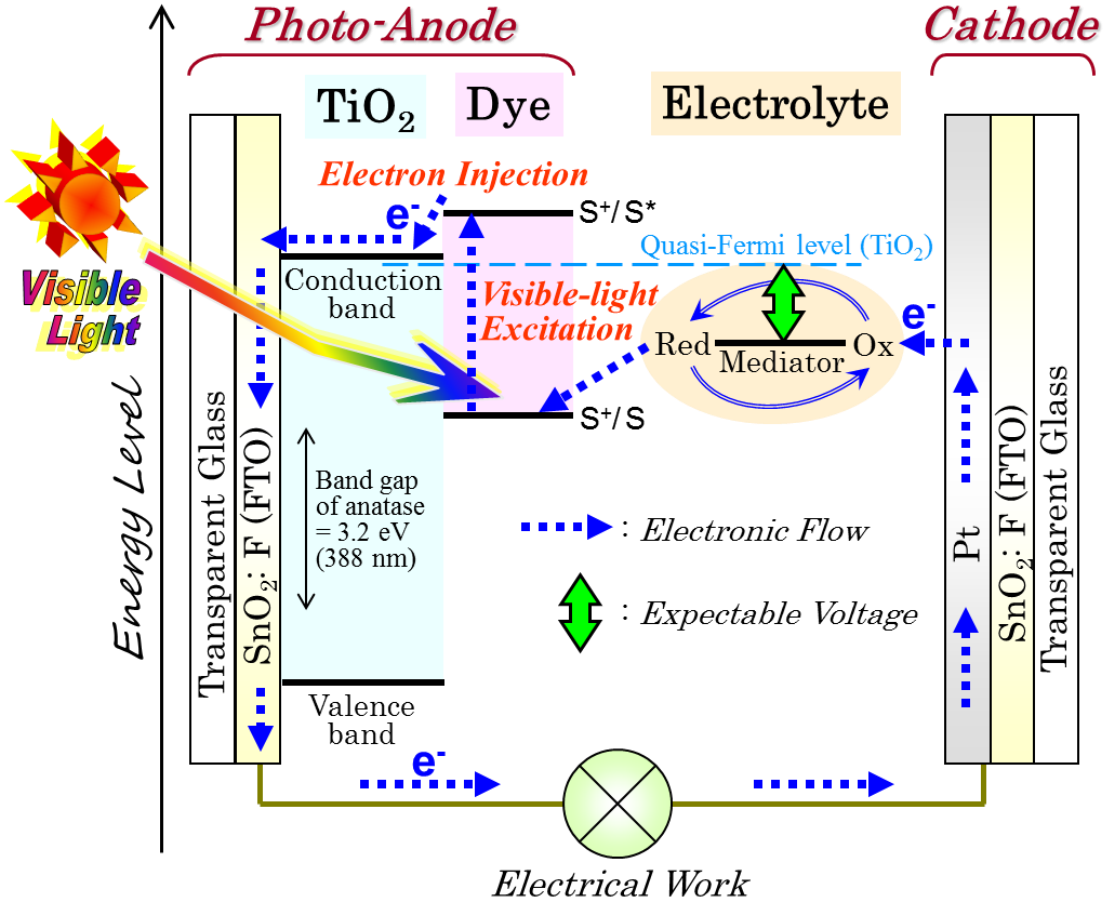

Fig. S2 Schematic drawing of the operation principle for a DSSC.

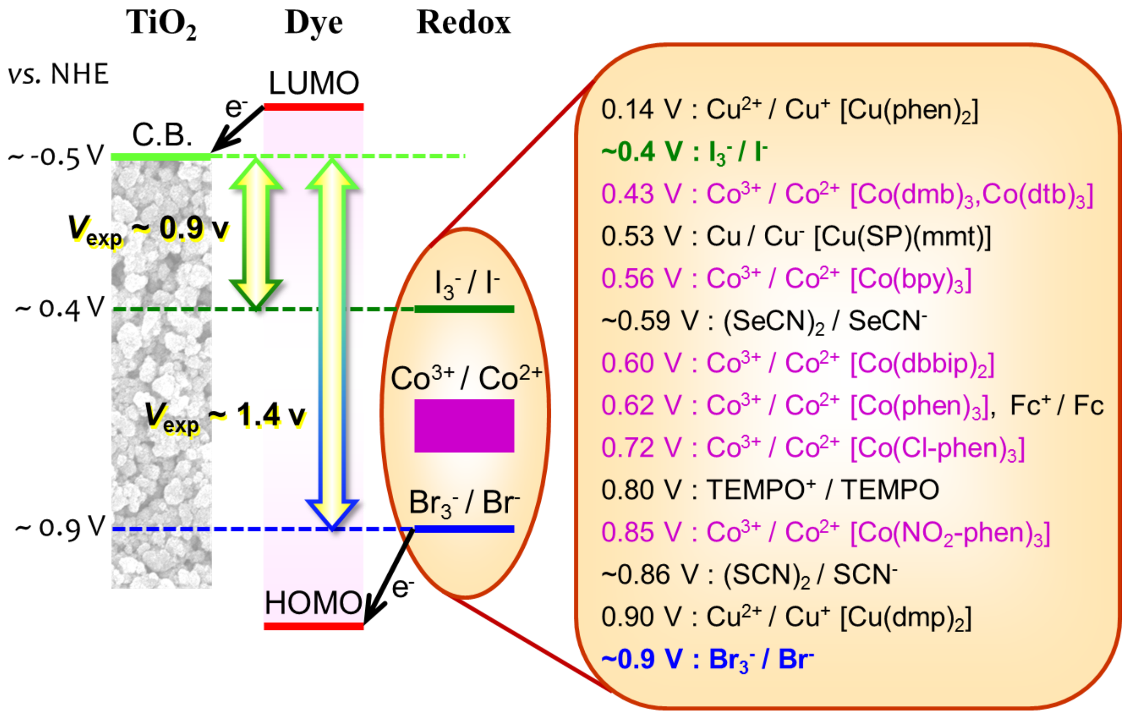

Fig. S3 Energy levels of representative redox mediators for DSSC electrolytes[14-16].

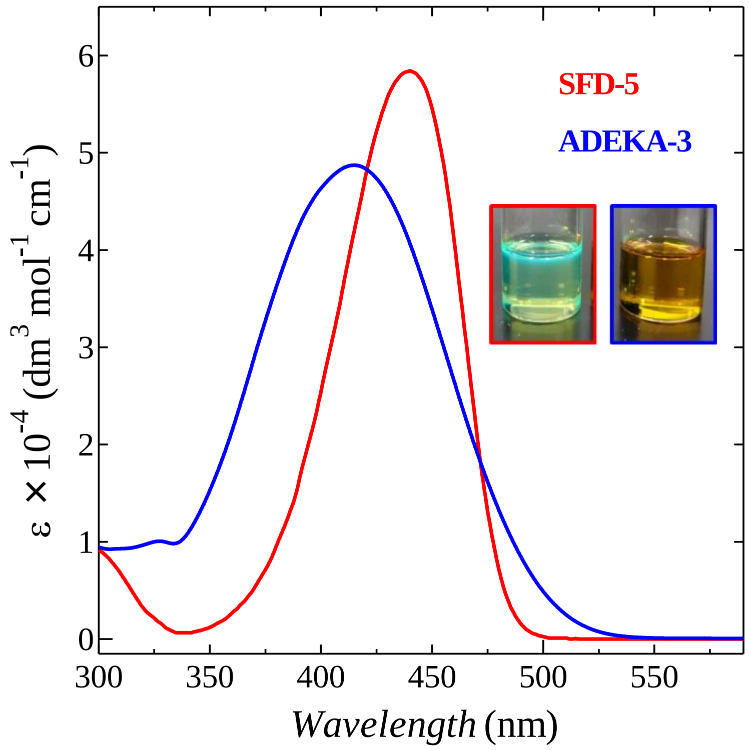

Fig. S4 UV-visible absorption spectra of **SFD-5** in an acetonitrile solution and **ADEKA-3** in a toluene solution. Insets show the photographs of the dye solutions.

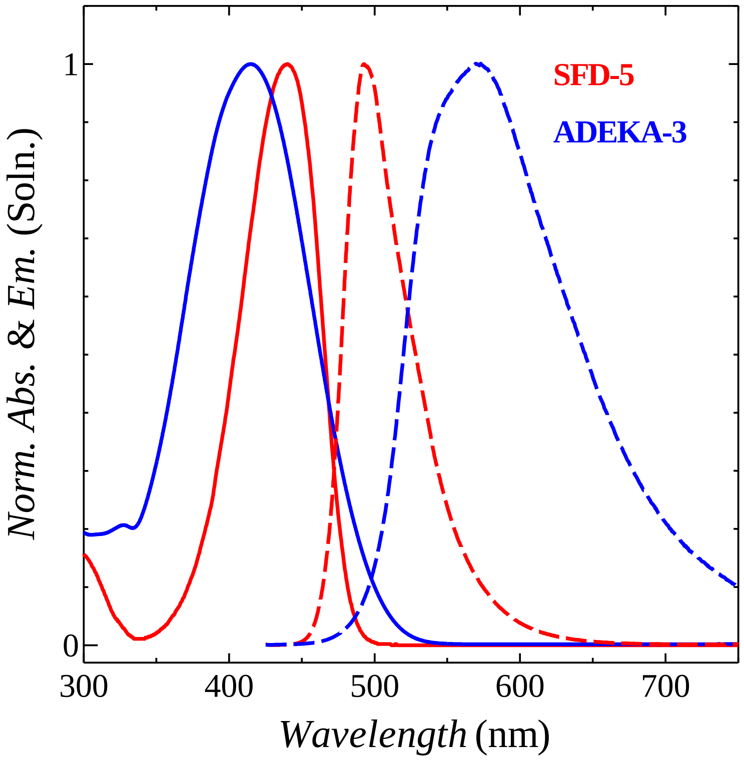

Fig. S5 Normalized UV-visible absorption spectra (solid lines) and emission spectra with λ_{ex} = 420 nm (dashed lines) of **SFD-5** in an acetonitrile solution and **ADEKA-3** in a toluene solution.

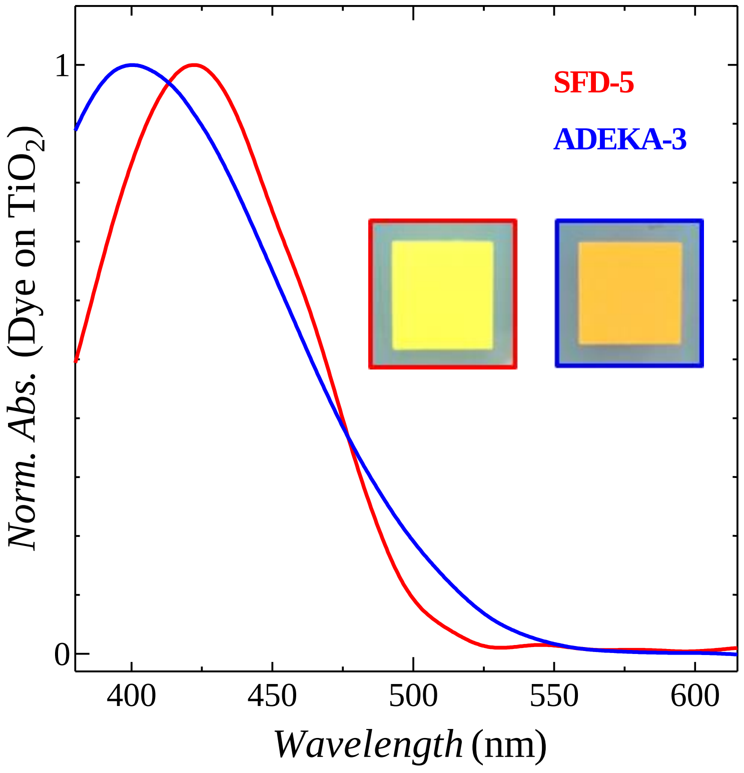

Fig. S6 Normalized Absorption spectra in visible region of **SFD-5** and **ADEKA-3** adsorbed on TiO_2 electrodes (t ~ 1 μm).

Table S1 Optical and electrochemical data for **SFD-5** and **ADEKA-3**.

Dye	Absorption[a]		Emission[a]	$E_{0\text{-}0}$ (eV)[b]	Potential *vs* NHE[c]		Driving Force[d]	
	λ_{max} (nm)	ε_{max} (dm^3 mol^{-1} cm^{-1})	λ_{max} (nm)	on TiO_2	E_{ox} (V)	E_{ox}^* (V)	$\lvert\Delta G_{inj}\rvert$ (eV)	$\lvert\Delta G_{reg}\rvert$ (eV)
**SFD-5**	440	58,400	493	2.48	1.39	-1.09	0.59	0.49
**ADEKA-3**	415	48,700	570	2.30	1.18	-1.12	0.62	0.28

a) Steady-state absorption and emission data were observed using acetonitrile (for **SFD-5**) and toluene (for **ADEKA-3**) as solvents. *b*) Lowest transition energies ($E_{0\text{-}0}$, approximately HOMO-LUMO gaps) were estimated from absorption onsets for the absorption spectra of the dye-adsorbed TiO_2 electrodes. *c*) Oxidation potentials (E_{ox} *vs*. NHE) were determined from cyclic voltammetry measurements. Excited state oxidation potentials (E_{ox}^* *vs*. NHE) were estimated from E_{ox} and $E_{0\text{-}0}$ ($E_{ox}^* = E_{ox} - E_{0\text{-}0}$). *d*) Driving forces for electron transfer processes. ΔG_{inj}: Driving forces for the electron injection from the singlet excited state (E_{ox}^*) of the dye to the TiO_2 conduction band (−0.5 V *vs*. NHE). ΔG_{reg}: Driving forces for the regeneration process of the dye radical cation state (E_{ox}) by the Br_3^-/Br^- redox state (+0.9 V *vs*. NHE).

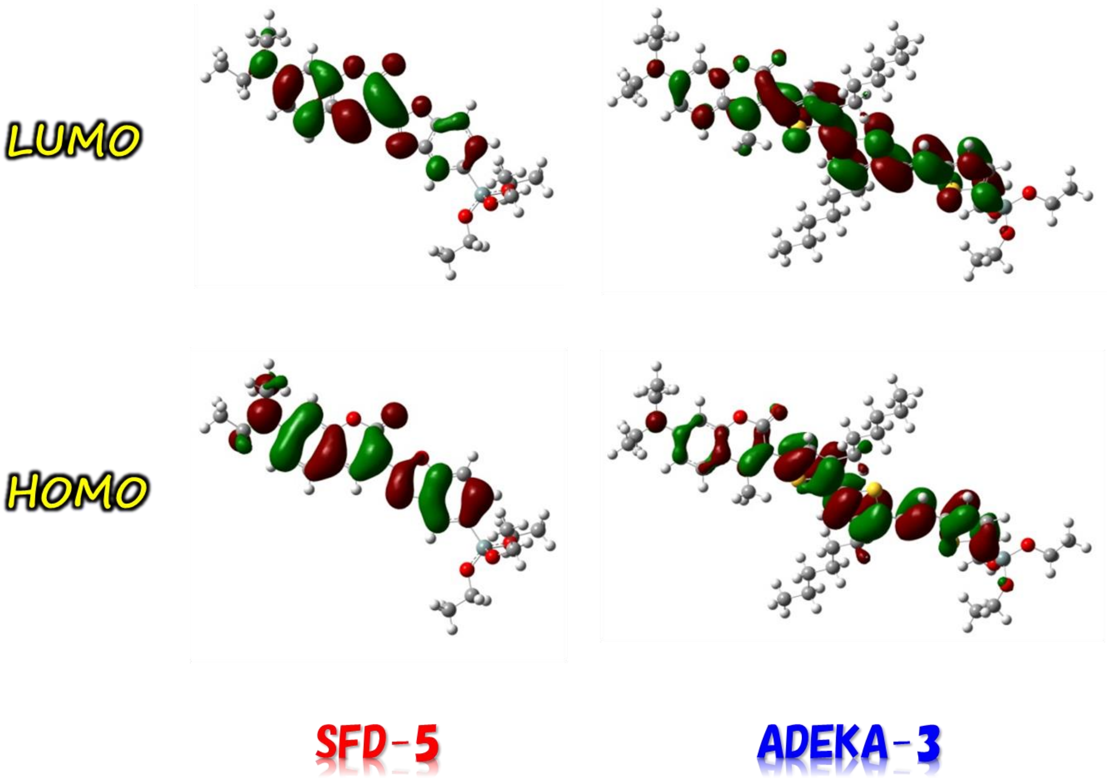

Fig. S7 Molecular orbitals of HOMO and LUMO for **SFD-5** and **ADEKA-3** calculated by DFT at the B3LYP/6-31+G(d,p) level.

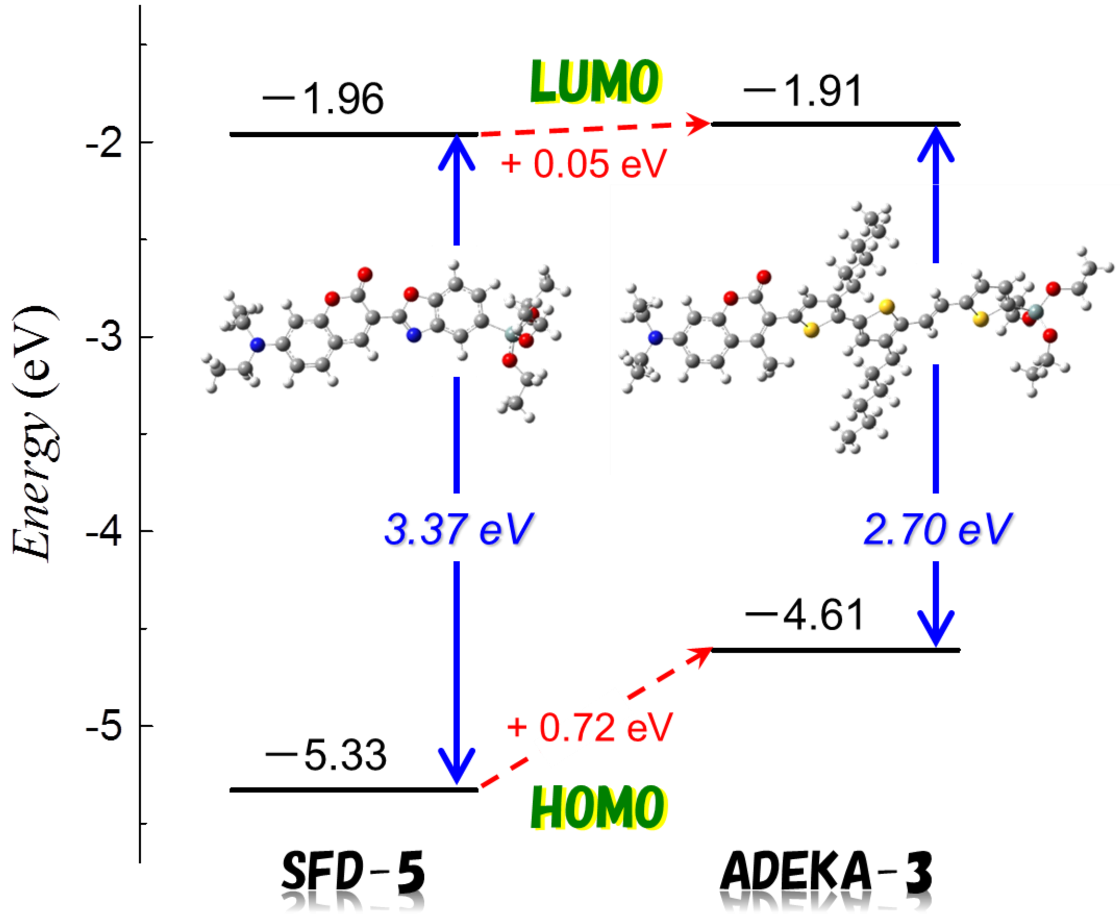

Fig. S8 Energy levels in frontier orbitals of HOMO and LUMO for **SFD-5** and **ADEKA-3** calculated by DFT at the B3LYP/6-31+G(d,p) level.

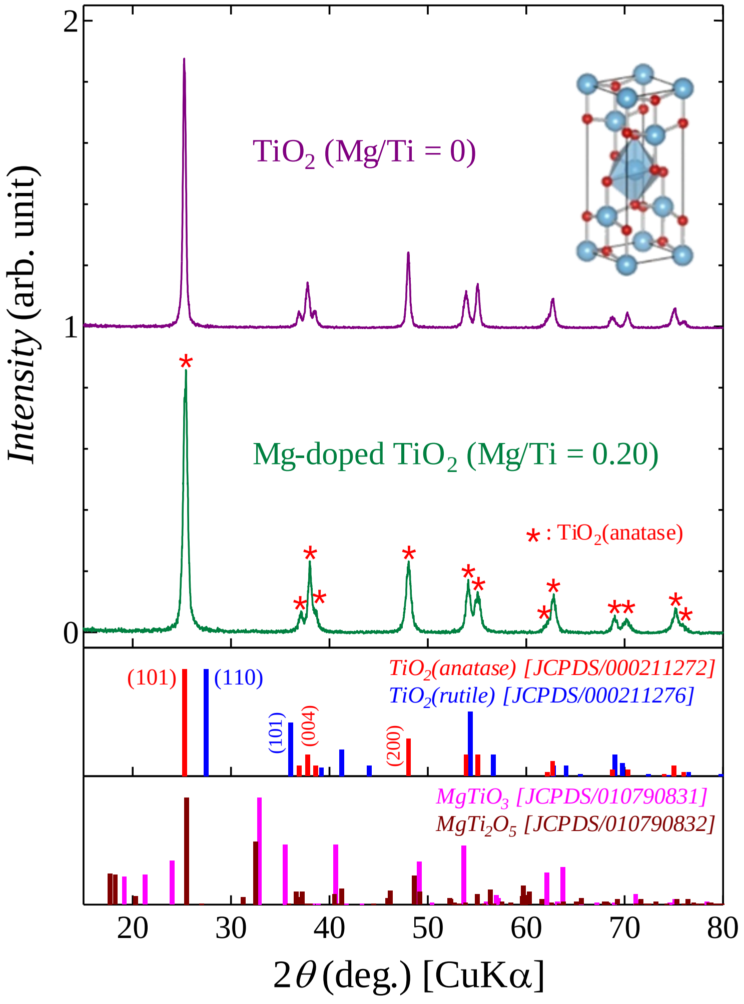

Fig. S9 XRD patterns at room temperature of the Mg-doped TiO_2 (Mg/Ti = 0.20, atomic ratio) and TiO_2 powders synthesized by the solvothermal method. The reported patterns for anatase-TiO_2, rutile-TiO_2, $MgTiO_3$, and $MgTi_2O_5$ are shown with several Miller indices.

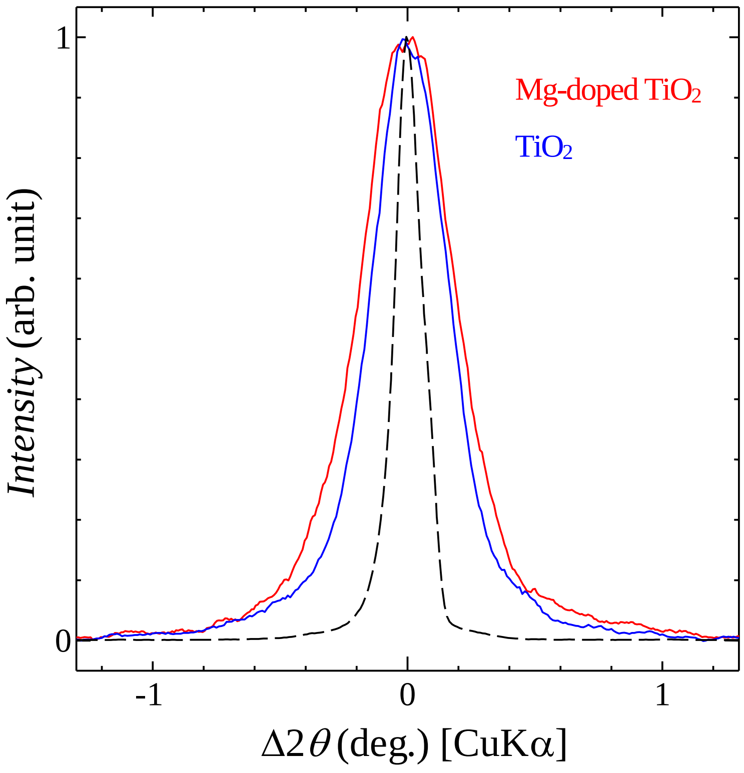

Fig. S10 The normalized XRD peaks of (101) plane for the Mg-doped TiO_2 (Mg/Ti = 0.20, atomic ratio) and TiO_2 powders synthesized by the solvothermal method. Dashed line represents the result of a diffraction peak of (111) plane for a standard Si crystal. The nanoparticle sizes of the Mg-doped TiO_2 and TiO_2 powders were estimated to be ~25 nm and ~27 nm, respectively, from the diffraction peak widths by using the Scherrer equation.

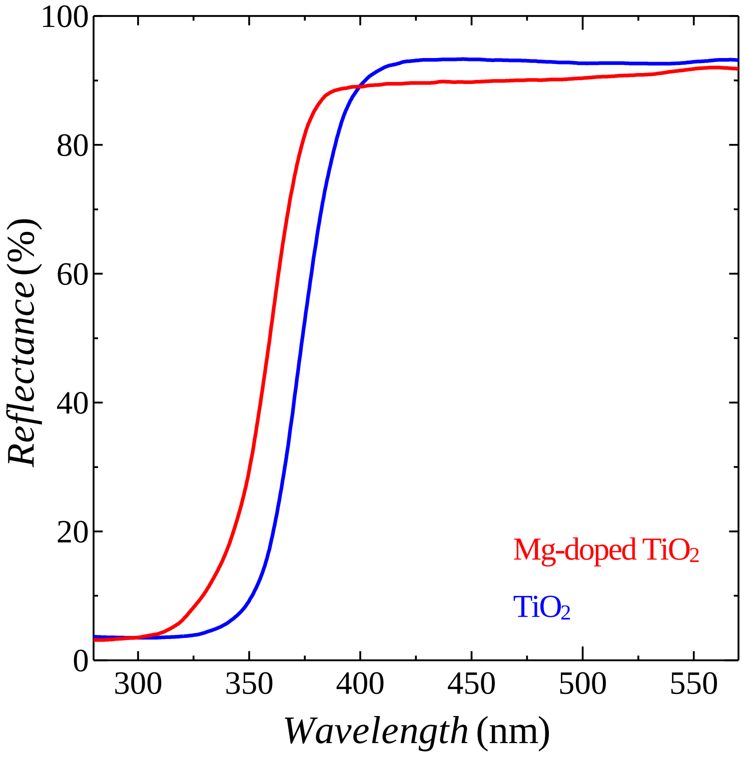

Fig. S11 UV-visible diffuse reflectance spectra of the Mg-doped TiO_2 (Mg/Ti = 0.20, atomic ratio) and TiO_2 powders synthesized by the solvothermal method.

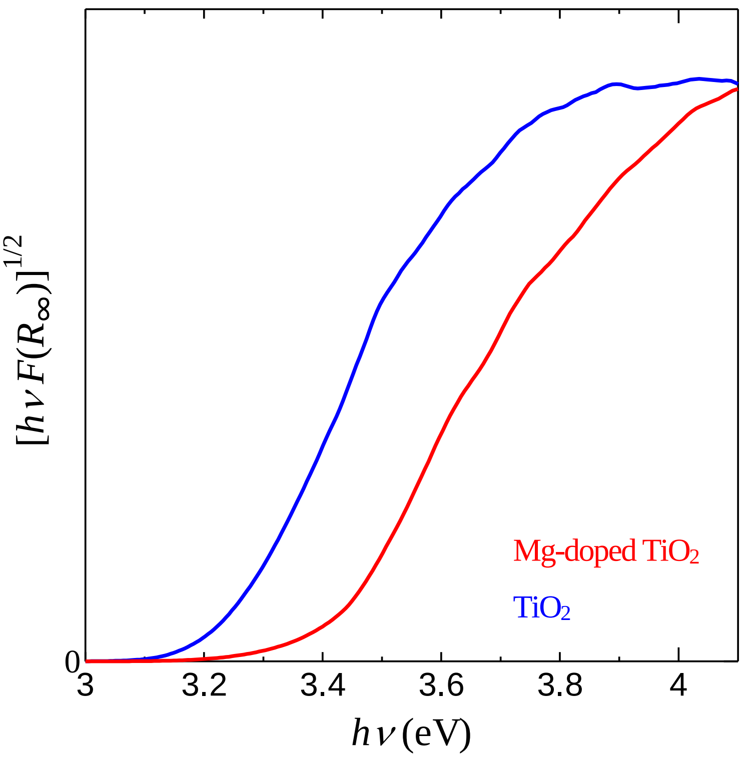

Fig. S12 Tauc plots obtained from the diffuse reflectance spectra of the Mg-doped TiO_2 (Mg/Ti = 0.20, atomic ratio) and TiO_2 powders synthesized by the solvothermal method.

Entry No. (dye, electrode, surface modification, electrolyte, temperature)

Entry 1 (**SFD-5**, TiO_2, none, Electrolyte A, 25 °C)

Entry 2 (**ADEKA-3**, TiO_2, none, Electrolyte A, 25 °C)

Entry 3 (**ADEKA-3**, Mg-doped TiO_2, none, Electrolyte A, 25 °C)

Entry 4 (**ADEKA-3**, Mg-doped TiO_2, MgO, Electrolyte A, 25 °C)

Entry 5 (**ADEKA-3,** Mg-doped TiO_2, MgO + Al_2O_3, Electrolyte A, 25 °C)

Entry 6 (**ADEKA-3,** Mg-doped TiO_2, MgO + Al_2O_3, Electrolyte B, 25 °C)

Entry 7 (**ADEKA-3,** Mg-doped TiO_2, MgO + Al_2O_3, Electrolyte C, 25 °C)

Entry 8 (**ADEKA-3,** Mg-doped TiO_2, MgO + Al_2O_3, Electrolyte C, 5 °C)

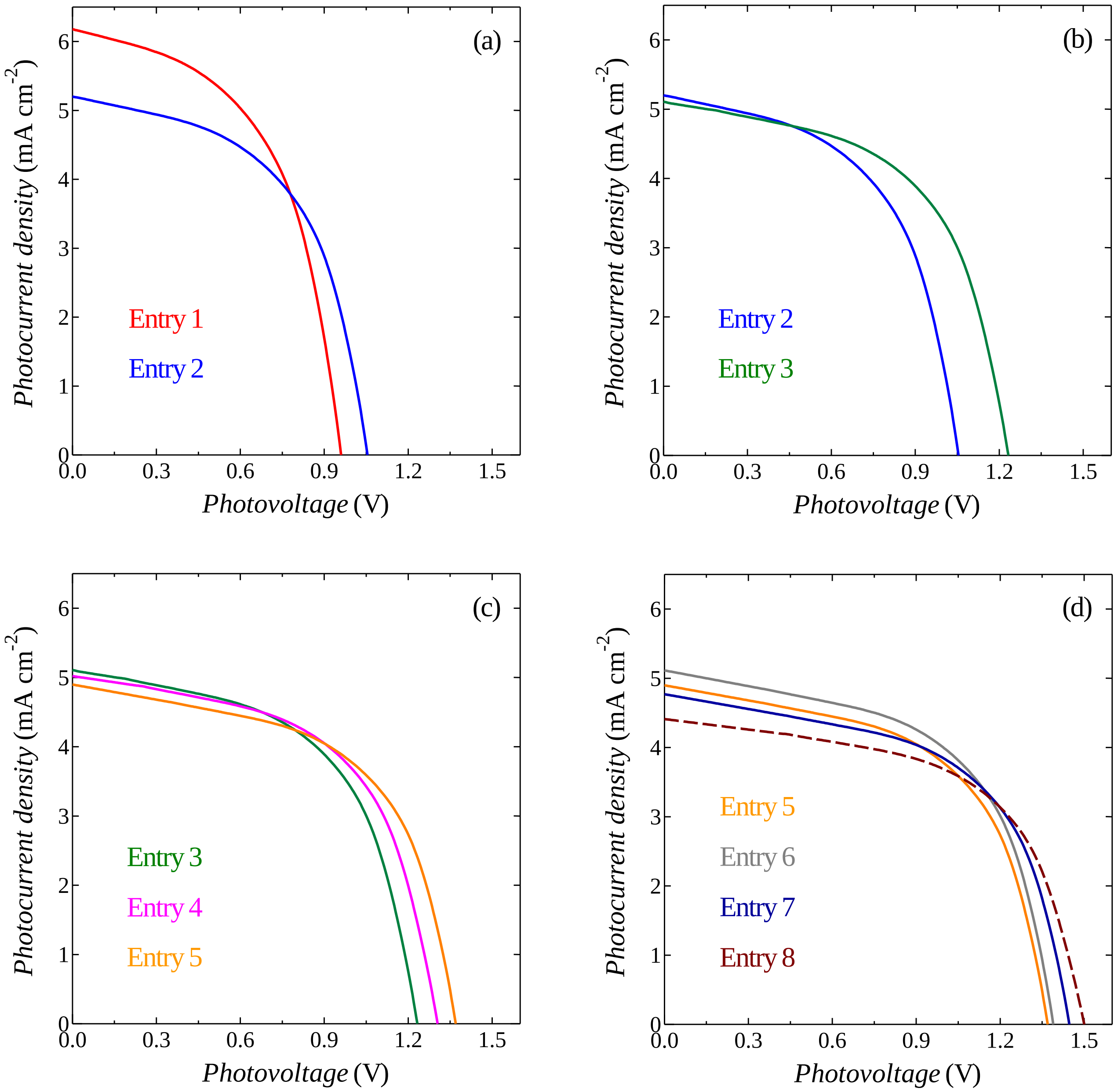

Fig. S13 *J-V* properties of the cells with the alkoxysilyl-anchor coumarin dye of **SFD-5** or **ADEKA-3**, with the TiO_2 or Mg-doped TiO_2 (Mg/Ti = 0.20, atomic ratio) electrode, without or with the surface modification of the Mg-doped TiO_2 electrode, and with the Br_3^-/Br^- redox electrolyte solution of Electrolyte A, B, or C under the simulated one sun irradiation (AM-1.5G, 100 mW cm^{-2}) at 25 or 5 ºC. The entry numbers correspond to those in Table 1.

Entry No. (dye, electrode, surface modification, electrolyte, temperature)
Entry 1 (**SFD-5**, TiO_2, none, Electrolyte A, 25 °C)
Entry 2 (**ADEKA-3**, TiO_2, none, Electrolyte A, 25 °C)

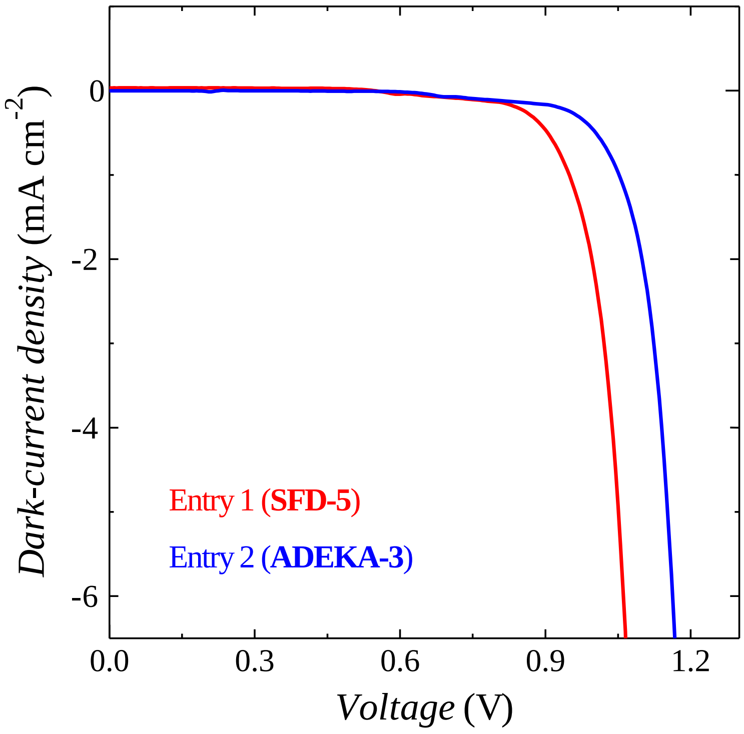

Fig. S14 Dark *J-V* properties of the cells with the TiO_2 electrodes sensitized by **SFD-5** and **ADEKA-3** and the Br_3^-/Br^- redox electrolyte solution of Electrolyte A (Entry 1 and 2 in Table 1).

Entry No. (dye, electrode, surface modification, electrolyte, temperature)
Entry 1 (**SFD-5**, TiO_2, none, Electrolyte A, 25 °C)
Entry 2 (**ADEKA-3**, TiO_2, none, Electrolyte A, 25 °C)

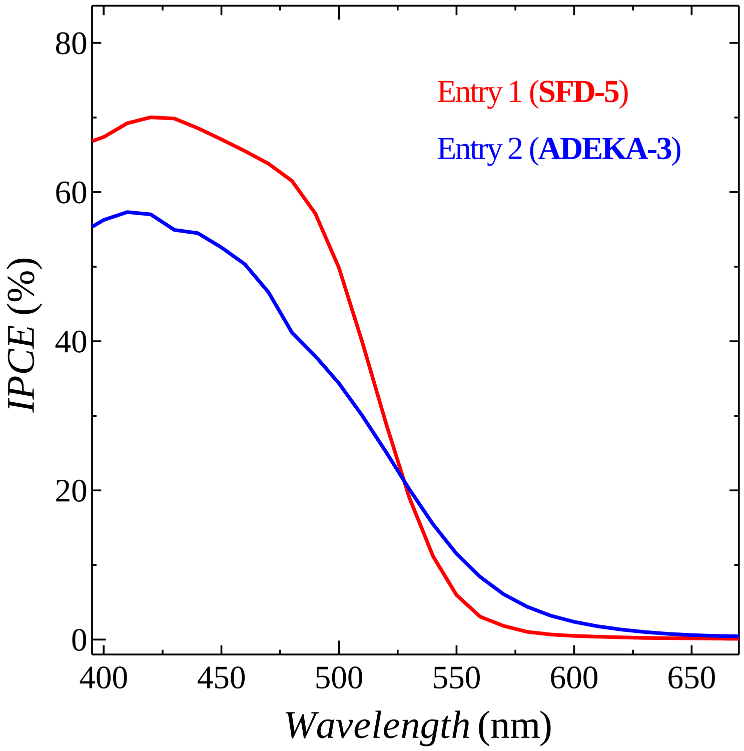

Fig. S15 *IPCE* spectra of the cells with the TiO_2 electrodes sensitized by **SFD-5** and **ADEKA-3** and the Br_3^-/Br^- redox electrolyte solution of Electrolyte A (Entry 1 and 2 in Table 1).

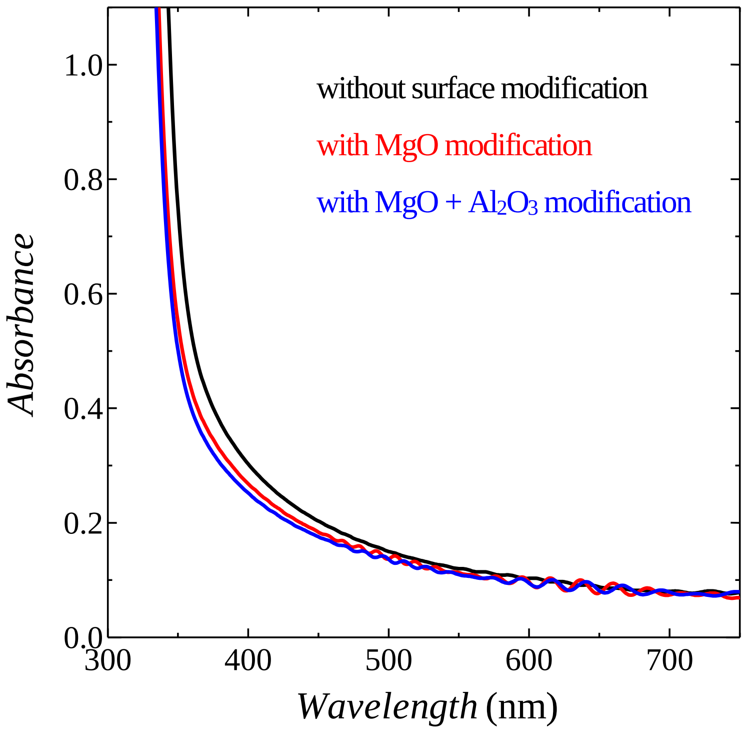

Fig. S16 UV-visible absorption spectra of the Mg-doped TiO_2 electrode without surface modification, the electrode with the MgO modification, and the electrode with the MgO + Al_2O_3 modification.

Table S2 Photovoltaic parameters of the cells with the alkoxysilyl-anchor coumarin dye of **ADEKA-3**, with the Mg-doped TiO_2 (Mg/Ti = 0.20, atomic ratio) electrode, with the surface modification of the Mg-doped TiO_2 electrode, and with the Br_3^-/Br^- redox electrolyte solution of Electrolyte C under the illumination of the simulated sunlight (AM-1.5G, 100 mW cm^{-2}) at 25 ºC: short-circuit photocurrent density (J_{sc}), open-circuit photovoltage (V_{oc}), fill factor (*FF*), and light-to-electric energy conversion efficiency (η).

Entry	Dye	Electrode	Surface Modification	Electrolyte	Temp.	J_{sc} (mA cm^{-2})	V_{oc} (V)	*FF*	η (%)
7a	**ADEKA-3**	Mg-doped TiO_2	$MgO + Al_2O_3$	C	25 ℃	4.77	1.45	0.56	3.9
7b	**ADEKA-3**	Mg-doped TiO_2	$MgO + Al_2O_3$	C	25 ℃	4.69	1.45	0.57	3.9
7c	**ADEKA-3**	Mg-doped TiO_2	$MgO + Al_2O_3$	C	25 ℃	4.90	1.46	0.55	3.9
Av.						4.79	1.45	0.56	3.9

Table S3a Reported photovoltaic parameters for single-DSSCs with V_{oc} of 0.8 V and upward.

Dye	Redox	J_{sc} (mA cm^{-2})	V_{oc} (V)	*FF*	η (%)	Year	Ref.
mercurochrome	I_3^-/I^-	1.40	0.85	0.66	0.94	2001	17
N719	I_3^-/I^-	17.73	0.846	0.745	11.18	2005	18
NKY-003	I_3^-/I^-	3.48	**1.00**	0.71	2.5	2008	1
YE05	I_3^-/I^-	17.0	0.80	0.74	10.1	2009	19
N719	I_3^-/I^-	1.68	0.872	0.69	1.0	2012	20
YF03	I_3^-/I^-	6.494	0.807	0.700	3.67	2012	21
MK-94	I_3^-/I^-	6.17	0.80	0.70	3.4	2014	22
TPA-TTAR-T-A (1)	I_3^-/I^-	7.66	0.946	0.658	4.76	2015	23
mercurochrome	Br_3^-/Br^-	0.29	0.99	0.46	0.16	2001	17
Eosin Y	Br_3^-/Br^-	2.72	0.823	0.657	1.5	2005	24
TC301	Br_3^-/Br^-	4.00	**1.156**	0.796	3.68	2010	25
SFD-5	Br_3^-/Br^-	1.80	**1.21**	0.55	1.2	2013	2
YD2-*o*-C8	$[Co(bpy)_3]^{3+/2+}$	17.3	0.965	0.71	11.9	2011	26
Y123	$[Co(bpy)_3]^{3+/2+}$	15.6	0.909	0.70	10.0	2012	27
MK-2	$[Co(bpy)_3]^{3+/2+}$	15.1	0.882	0.75	10.0	2013	28
MK-33	$[Co(bpy)_3]^{3+/2+}$	11.7	0.825	0.69	6.6	2013	29
MK-34	$[Co(bpy)_3]^{3+/2+}$	14.4	0.838	0.76	9.2	2013	30
JK-306	$[Co(bpy)_3]^{3+/2+}$	14.83	0.95	0.71	10.02	2013	31
C259 + C239	$[Co(bpy)_3]^{3+/2+}$	17.85	0.891	0.722	11.5	2013	32
JF419	$[Co(bpy)_3]^{3+/2+}$	16.2	0.840	0.76	10.3	2013	33
YA422	$[Co(bpy)_3]^{3+/2+}$	16.25	0.890	0.737	10.65	2014	34
WW-6	$[Co(bpy)_3]^{3+/2+}$	17.69	0.809	0.735	10.5	2014	35
SM315	$[Co(bpy)_3]^{3+/2+}$	18.1	0.91	0.78	13.0	2014	36
GY50	$[Co(bpy)_3]^{3+/2+}$	18.53	0.885	0.773	12.75	2014	37
Y350-OC12	$[Co(bpy)_3]^{3+/2+}$	16.0	0.968	0.74	11.5	2014	38
MK-88	$[Co(bpy)_3]^{3+/2+}$	10	0.806	0.70	5.8	2014	39
C235	$[Co(phen)_3]^{3+/2+}$	14.6	0.93	0.743	10.1	2012	40
C239	$[Co(phen)_3]^{3+/2+}$	6.91	**1.05**	0.76	5.5	2012	41
C272 + YD2-o-C8	$[Co(phen)_3]^{3+/2+}$	15.52	0.907	0.762	10.7	2015	42
C275	$[Co(phen)_3]^{3+/2+}$	17.03	0.956	0.770	12.5	2015	43
ADEKA-1 + LEG4	$[Co(phen)_3]^{3+/2+}$	17.77	**1.018**	0.765	13.8	2015	44

Table S3b Reported photovoltaic parameters for single-DSSCs with V_{oc} of 0.8 V and upward.

Dye	Redox	J_{sc} (mA cm^{-2})	V_{oc} (V)	*FF*	η (%)	Year	Ref.
D35	**[Co(NO$_2$-phen)$_3$]$^{3+/2+}$**	4.38	**1.03**	0.51	2.29	2011	16
D35	**[Co(Cl-phen)$_3$]$^{3+/2+}$**	6.35	**1.01**	0.56	3.57	2011	16
ADEKA-1	**[Co(Cl-phen)$_3$]$^{3+/2+}$**	15.6	**1.036**	0.774	12.5	2014	45
ADEKA-1 + SFD-5	**[Co(Cl-phen)$_3$]$^{3+/2+}$**	16.0	**1.034**	0.774	12.8	2015	46
D35	**[Co(py-pz)$_3$]$^{3+/2+}$**	2.5	0.90	0.66	1.5	2013	47
D35	**[Co(Mepy-pz)$_3$]$^{3+/2+}$**	0.78	0.88	0.58	0.4	2013	47
Y123	**[Co(bpy-pz)$_2$]$^{3+/2+}$**	13.06	0.998	0.774	10.08	2012	48
D35	**[Co(bpy-pz)$_2$]$^{3+/2+}$**	5.3	**1.02**	0.68	3.6	2013	47
D35	**[Co(Me$_2$bpy-pz)$_2$]$^{3+/2+}$**	6.1	**1.02**	0.61	3.7	2013	47
Z907	**[Co(dbbip)$_2$]$^{3+/2+}$**	8.40	0.84	0.56	3.9	2003	49
Y123	**[Co(terpy)$_2$]$^{3+/2+}$**	13.04	0.893	0.64	7.4	2013	50
Y123	**[Co(Cl-terpy)$_2$]$^{3+/2+}$**	13.7	0.922	0.68	8.7	2013	50
MK-2	**[Co(PY5Me$_2$)]$^{3+/2+}$**	8.1	0.993	0.76	6.1	2012	51
C218	**[Cu(dmp)$_2$]$^{2+/+}$**	11.29	0.932	0.66	7.0	2011	52
Carbz-PAHTDTT	**Fc$^+$/Fc**	12.2	0.842	0.73	7.5	2011	53
D149	**TEMPO$^+$/TEMPO**	6.9	0.860	0.67	3.9	2008	54
D131	**R-TEMPO$^+$/R-TEMPO**	2.7	0.860	0.65	1.5	2010	55
1-amino-4-hydroxyanthraquinone	**perylene**	2.3	**1.18**	0.45	1.22	2012	56
1,2,3,4,5-pentamethylcyclopentadiene	**perylene**	2.64	**1.21**	0.527	1.68	2014	57
K68	**spiro-OMeTAD**	5.7	0.8967	0.761	3.88	2009	58
JK2	**spiro-OMeTAD**	3.85	**1.0875**	0.677	3.17	2009	58
Z907	**spiro-MeOTAD**	6.45	0.849	0.683	3.7	2011	59
TA-St-CA	**spiro-MeOTAD**	4.25	**1.020**	0.689	3.0	2011	59
MK-2	**spiro-MeOTAD**	8.1	0.815	0.61	4.0	2012	60
LEG4	**spiro-OMeTAD**	9.70	0.90	0.66	5.8	2013	61

Table S4 Reported photovoltages of various single solar cells[62].

Solar Cell	V_{oc} (V)
Si (crystalline)	0.740
Si (multicrystalline)	0.663
Si (thin transfer submodule)	0.687
Si (thin-film minimodule)	0.492
Si (amorphous)	0.896
Si (microcrystalline)	0.548
GaAs (thin film)	1.122
GaAs (multicrystalline)	0.994
InP (crystalline)	0.878
GaInP	1.455
CIGS (cell)	0.757
CIGS (thin film)	0.746
CIGS (minimodule)	0.701
CIGSS (Cd free)	0.686
CdTe (cell)	0.876
CdTe (thin film)	0.877
CZTSS (thin film)	0.513
CZTS (thin film)	0.701
DSSC	0.744
DSSC (minimodule)	0.754
DSSC (submodule)	0.697
OPV	0.867
OPV (minimodule)	0.686
PSC	1.090

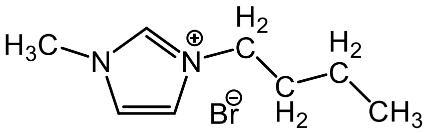

BMImBr

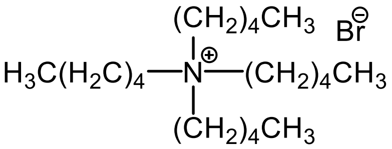

TPABr

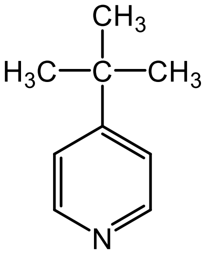

TBP

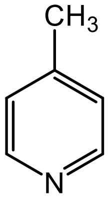

MP

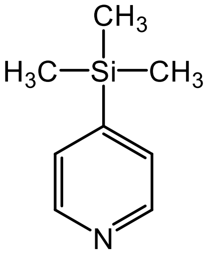

TMSP

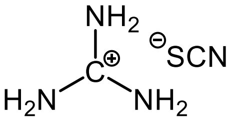

GuSCN

Fig. S17 Molecular structures of additives (1-*n*-butyl-3-methylimidazolium bromide: BMImBr, tetra-*n*-pentylammonium bromide: TPABr, 4-*tert*-butylpyridine: TBP, 4-methylpyridine: MP, 4-trimethylsilylpyridine: TMSP, guanidinium thiocyanate: GuSCN) used in the Br_3^-/Br^- redox electrolyte solutions.

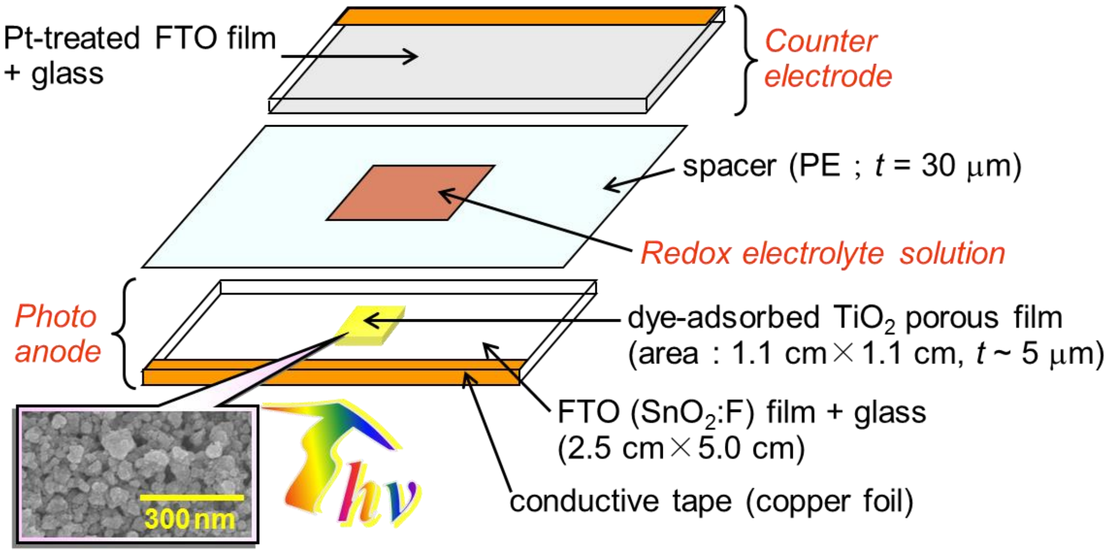

Fig. S18 Schematic drawing of the electrochemical cell of the open sandwich type used in this work.

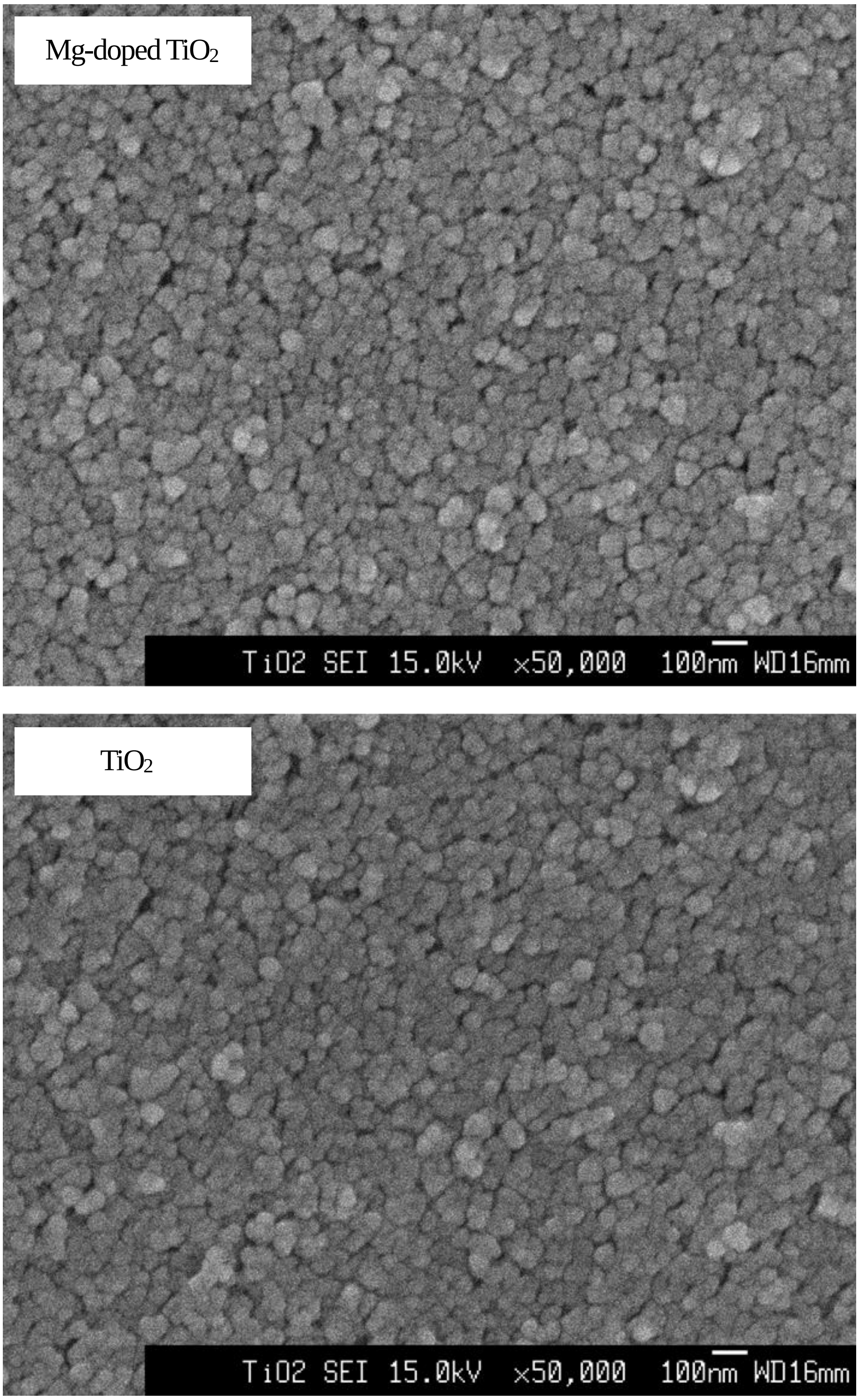

Fig. S19 SEM images of the Mg-doped TiO_2 (Mg/Ti = 0.20, atomic ratio) and TiO_2 electrodes.

References (Supporting Information)

1. Iwamoto, S. et al. *ChemSusChem* **1**, 401-403 (2008).
2. Kakiage, K. et al. *Chem. Commun.* **49**, 179-180 (2013).
3. Cullity, B. D. *Elements of X-ray diffraction*, Addision-Wesley Pub. Co., Reading, Mass., 2nd edn., 1978, ch. 3.
4. West, A. R. *Solid State Chemistry and its Applications*, John Wiley & Sons, Chichester, 1984, ch. 5.
5. Kakiage, K., Ishiuchi, S., Kyomen, T. & Hanaya, M. *Key Eng. Mater.* **534**, 40-45 (2013).
6. Tauc, J., Grigorovici, R. & Vancu, A. *Phys. Stat. Sol.* **15**, 627-637 (1966).
7. Tauc, J. in *Optical properties of solids*, ed. F. Abelès, North-Holland, Amsterdam, 1972.
8. Irie, H., Watanabe, Y. & Hashimoto, K. *J. Phys. Chem. B* **107**, 5483-5486 (2003).
9. Ito, S. et al. *Thin Solid Films* **516**, 4613-4619 (2008).
10. Xia, J. & Yanagida, S. *Solar Energy* **85**, 3143-3159 (2011).
11. Gaussian 09, Revision D.01, Frisch, M. J. et al. Gaussian, Inc., Wallingford CT (2009).
12. Lee, C., Yang, W. & Parr, R. G. *Phys. Rev. B* **37**, 785 (1988).
13. Becke, A. D. *J. Chem. Phys.* **98**, 5648-5652 (1993).
14. Wang, M., Grätzel, C., Zakeeruddin, S. M. & Grätzel, M. *Energy Environ. Sci.* **5**, 9394-9405 (2012).
15. Cong, J., Yang, X., Kloo, L. & Sun, L. *Energy Environ. Sci.* **5**, 9180-9194 (2012).
16. Feldt, S. M., Wang, G., Boschloo, G. & Hagfeldt, A. *J. Phys. Chem. C* **115**, 21500-21507 (2011).
17. Hara, K. et al. *Sol. Energy Mater. Sol. Cells* **70**, 151-161 (2001).
18. Nazeeruddin, M. K. et al. *J. Am. Chem. Soc.* **127**, 16835-16847 (2005).
19. Bessho, T. et al. *J. Am. Chem. Soc.* **131**, 5930-5934 (2009).
20. Huang, F. et al. *J. Mater. Chem.* **22**, 17128-17132 (2012).
21. Yang, F. et al. *J. Mater. Chem.* **22**, 22550-22557 (2012).
22. Murakami, T. N., Yoshida, E. & Koumura, N. *Electrochim. Acta* **131**, 174-183 (2014).
23. Zhou, N. et al. *J. Am. Chem. Soc.* **137**, 4414-4423 (2015).
24. Wang, Z.-S., Sayama, K. & Sugihara, H. *J. Phys. Chem. B* **109**, 22449-22455 (2005).
25. Teng, C. et al. *Org. Lett.* **11**, 5542-5545 (2009).
26. Yella, A. et al. *Science* **334**, 629-634 (2011).
27. Tsao, H. N., Comte, P., Yi, C. & Grätzel, M. *ChemPhysChem* **13**, 2976-2981 (2012).

28. Xiang, W., Huang, W., Bach, U. & Spiccia, L. *Chem. Commun.* **49**, 8997-8999 (2013).

29. Murakami, T. N. et al. *J. Mater. Chem. A* **1**, 792-798 (2013).

30. Uchiyama, T. et al. *Chem. Lett.* **42**, 453-454 (2013).

31. Lim, K. et al. *ChemSusChem* **6**, 1425-1431 (2013).

32. Zhang, M. et al. *Energy Environ. Sci.* **6**, 2944-2949 (2013).

33. Yella, A. et al. *Chem. Mater.* **25**, 2733-2739 (2013).

34. Yang, J. et al. *J. Am. Chem. Soc.* **136**, 5722-5730 (2014).

35. Luo, J. et al. *J. Am. Chem. Soc.* **136**, 265-272 (2014).

36. Mathew, S. et al. *Nat. Chem.* **6**, 242-247 (2014).

37. Yella, A. et al. *Angew. Chem. Int. Ed.* **53**, 2973-2977 (2014).

38. Yi, C. et al. *ChemSusChem* **7**, 1107-1113 (2014).

39. Murakami, T. N., Koumura, N., Kimura, M. & Mori, S. *Langmuir* **30**, 2274-2279 (2014).

40. Cao, Y. et al. *Phys. Chem. Chem. Phys.* **14**, 8282-8286 (2012).

41. Xu, M. et al. *Chem. Sci.* **3**, 976-983 (2012).

42. Yao, Z. et al. *Energy Environ. Sci.* **8**, 1438-1442 (2015).

43. Yao, Z. et al. *J. Am. Chem. Soc.* **137**, 3799-3802 (2015).

44. Kakiage, K. et al. *Chem. Commun.* **51**, 15894-15897 (2015).

45. Kakiage, K. et al. *Chem. Commun.* **50**, 6379-6381 (2014).

46. Kakiage, K. et al. *Chem. Commun.* **51**, 6315-6317 (2015).

47. Feldt, S. M. et al. *Phys. Chem. Chem. Phys.* **15**, 7087-7097 (2013).

48. Yum, J.-H. et al. *Nat. Commun.* **3**, 631 (2012).

49. Nusbaumer, H., Zakeeruddin, S. M., Moser, J.-E. & Grätzel, M. *Chem. Eur. J.* **9**, 3756-3763 (2003).

50. Aribia, K. B., Moehl, T., Zakeeruddin, S. M. & Grätzel, M. *Chem. Sci.* **4**, 454-459 (2013).

51. Kashif, M. K. et al. *J. Am. Chem. Soc.* **134**, 16646-16653 (2012).

52. Bai, Y. et al. *Chem. Commun.* **47**, 4376-4378 (2011).

53. Daeneke, T. et al. *Nat. Chem.* **3**, 211-215 (2011).

54. Zhang, Z. et al. *Adv. Funct. Mater.* **18**, 341-346 (2008).

55. Kato, F. et al. *Chem. Lett.* **39**, 464-465 (2010).

56. Ishii, A. & Miyasaka, T. *Chem. Commun.* **48**, 9900-9902 (2012).

57. Ishii, A. & Miyasaka, T. *ChemPhysChem* **15**, 1028-1032 (2014).

58. Chen, P. et al. *Nano Lett.* **9**, 2487-2492 (2009).

59. Jang, S.-R. et al. *ACS Nano* **5**, 8267-8274 (2011).

60. Cappel, U. B., Daeneke, T. & Bach, U. *Nano Lett.* **12**, 4925-4931 (2012).

61. Xu, B. et al. *J. Mater. Chem. A* **1**, 14467-14470 (2013).

62. Green, M. A. et al. *Prog. Photovolt: Res. Appl.* **23**, 805 (2015).

Acknowledgments

This work was partly supported by the “Element Innovation” Project by Ministry of Education, Culture, Sports, Science & Technology in Japan and by JSPS KAKENHI Grant Number 15H03848.
